# A Scoping Review of Comparative Healthcare Codes of Ethics Studies

**DOI:** 10.1111/jan.16857

**Published:** 2025-03-03

**Authors:** Ryan Essex, Lydia Mainey, Francine Gonzales‐Walters, Phil Gurnett, Sharon Marie Weldon

**Affiliations:** ^1^ Institute for Lifecourse Development University of Greenwich London UK; ^2^ School of Nursing, Midwifery and Social Sciences Central Queensland University Rockhampton Queensland Australia; ^3^ Barts Health NHS Trust London UK; ^4^ Imperial College London London UK

**Keywords:** codes, codes of ethics, comparative study, scoping review

## Abstract

**Background:**

Codes of ethics are, for many, important documents that define the key values and behaviours expected of healthcare professionals. They are also documents that have been widely criticised. These criticisms range from being vague to failing to provide guidance on many important issues. Codes, however, vary substantially in their scope, content and the guidance they provide.

**Aim:**

This scoping review sought, in the context of comparative studies of codes, to examine the form (i.e., the structure of the code, its contents, principles or rules for example) and function (what the code says it does, either explicitly or implicitly) of codes, along with their points of con/divergence.

**Method:**

A systematic search was carried out using Scopus, PsycInfo, CINAHL and Medline.

**Findings:**

Thirty‐one papers met inclusion criteria and were included in this review. Results suggest that while there were a number of similarities seen across codes, there were also substantial points of divergence related to the content of codes and structure. These differences were seen across professions, countries and time, suggesting that culture, history, politics and perhaps even geography influence the content of codes.

**Discussion:**

These findings are discussed in light of the broader literature that examines and critiques codes.

## 1 Introduction

Codes of ethics have existed for over 200 years in various forms (Patuzzo et al. 2018). Over the last 50 years, codes have proliferated and become ubiquitous in healthcare. While no official count exists, there are likely to be hundreds, if not thousands, of codes globally that are related to healthcare. These codes come from international (Internatioal Council of Nurses 2021), national (Medical College of Chile 2022) and sub‐national bodies (College of Nurses of Ontario 2023). They can be developed by regulatory bodies or healthcare unions and can cover entire professions or may focus on sub‐specialities or specific clinical settings, like schools, for example (The American Correctional Health Services Association 1996). What codes ‘do’ also varies substantially. While primarily seen as tools to guide behaviour or at least broad standards by which professions should conduct themselves, codes can also serve a range of other purposes, from establishing and re‐enforcing professional status (Linker 2005) to providing information to the public. In achieving these things, codes may or may not have regulatory force. Their content also varies substantially, from specific rules and guidance to more ambitious and aspirational goals (Banks 2003). To add further complexity to this picture, codes of conduct and codes of practice may also contain ethical content and guidance. In this paper, we limit our discussion to self‐identified codes of ethics; however, we conceptualise codes of ethics in their broadest sense, regardless of their function or form.

While most professional and regulatory bodies will stress the importance and necessity of their code, codes have also been criticised for a range of reasons. These criticisms include the meta‐ethical^1^ claims that codes make (O'Donohue 2020; Snelling 2016), the fact they include or exclude specific content, like statements related to human rights (Tisdale and Symenuk 2020) or the fact that they are simply tools for professions to establish or entrench their power (Linker 2005). Perhaps most prominent amongst these criticisms, however, are concerns about the guidance (or lack thereof) that codes provide (Pattison and Wainwright 2010), with a number of authors arguing that codes simply cannot guide ethical behaviour (Dahnke 2009; Snelling 2017). These criticisms are, of course, closely tied to our expectations about the function and form of codes, that is, what we think codes are and what they should do. For example, if we see codes as a means to set broad behavioural expectations or set aspirational standards, rather than provide specific guidance in relation to behaviour, we may view criticisms about a code's ability to guide behaviour as misguided or unfair (Banks 1998). The criticisms we find largely speak to what it is we think codes do and, ultimately, what they should be.

A number of empirical studies of codes add further reason to be concerned about the form and function of codes and how health professionals are utilising them. In a scoping review that included 25 papers carried out by Collings‐Hughes et al. (2022), it was found that despite having knowledge of their existence and valuing codes, health professionals were largely unaware of the specific content of their respective codes. A study by Blackwood and Chiarella (2020) examined barriers to the ‘uptake and use’ of codes amongst nurses. This study found that in adopting codes, nurses faced a range of individual, professional, cultural and educational barriers that influenced their ability to engage with and apply their code of ethics.

Regardless of what it is the codes do, it is clear that they matter. At a very minimum, they set expectations for the profession, defining normative expectations, aims or aspirations. While there is some reason to be sceptical that codes can or do guide behaviour; there have been very real implications when it comes to seemingly small revisions of codes. For example, the American Psychological Society revised its code of ethics in 2002 in the wake of the September 11 attacks in the US. The move to direct Psychologists to ‘adhere to the requirements of the law, regulations, or other governing legal authority’ when facing a conflict with their ‘ethical responsibilities’ was criticised as a way to enable and legitimise Psychologists who were involved in the interrogation and torture of prisoners held in Guantanamo Bay and other military prisons (Pope 2011). While this is a somewhat extreme example, the details in codes are often hotly debated, and these do matter. At present, the American Nursing Association is reviewing its code of ethics. One point of particular contention has been about the responsibility of nurses to maintain care during strike action (Thomas 2024). The wording of the code is not an abstract concern; the US has witnessed multiple and often protracted nursing strikes over the last few years (Sharma et al. 2024).

One potential way to shed light on some of these issues above that has too often been overlooked is the utilisation of ‘comparative’ studies. That is studies that compare codes of ethics across time, place and profession to explore points of con/divergence and the practical implications of this. Such studies have the potential to answer a range of questions. For example, the distinct nature of nursing and medical ethics has long been debated (Grace 2024); comparative debates provide a means to examine how the respective professions approach ethics, but not only this, how we may also bridge divides between the professions when it comes to ethics, teamwork and communication, amongst other issues. Also widely discussed has been the influence of culture on ethics (Chukwuneke et al. 2014). Here comparative studies could play a role in informing discussions about decolonisation. When it comes to nursing, for example, Johnstone (2015) argues that while nursing ethics is ‘portrayed as universally applicable in all cultural contexts’, its ‘values, concepts and theories that underpin contemporary nursing ethics are very much a product of Western cultures’. Can the same be said about codes of ethics? It seems a reasonable starting point to examine, compare and even potentially work toward decolonising nursing codes by examining not only how and why codes differ but also the normative implications of this. Beyond these considerations, such an approach may shed light on other aspects of codes, how they shift over time and provide insight into the social and political forces that have shaped them.

This paper seeks to add to the above literature by scoping, mapping and synthesising the evidence that comes from comparative studies of codes of ethics; that is, studies that compare codes of ethics with other documents or against other codes. Specifically, we were interested in what these studies say about codes' form (i.e., the structure of the code, its contents, principles or rules, for example) and function (what the code says it does, either explicitly or implicitly) and points of con/divergence. In addition to informing debates about decolonisation, ethical imperialism and interprofessional differences, such studies have the potential to tell us a great deal about codes themselves, as well as their temporospatial similarities and differences.

## 2 Methods

### 2.1 Design

To map codes of ethics' form, function and points of con/divergence a scoping review was selected. Scoping reviews are particularly well suited for broad research questions across a breadth of evidence sources (Peters et al. 2021). Arksey and O'Malley's (2005) five‐stage methodological framework for scoping reviews was used to identify and analyse the literature. These five stages are: (1) identification of area of interest/research question, (2) literature search, (3) study selection/eligibility, (4) data extraction and (5) data synthesis and write‐up. Our search and reporting are consistent with PRISMA‐ScR guidance (Tricco et al. 2018). Each step of this process is outlined below.

### 2.2 Search Methods

Search terms were developed to capture the two key concepts, namely codes of ethics and the healthcare professions. To develop this search strategy, PRESS guidelines were consulted (McGowan et al. 2016), and a number of preliminary searches were carried out until we were confident our search was comprehensive. We limited our search terms for healthcare professions to those registered/regulated in the UK by the General Medical Council (physicians), the Nursing and Midwifery Council (nursing and midwifery) and the Health and Care Professions Council (15 allied health professions). We used common spelling and terminology variations; we also used a number of more general terms like ‘allied health’ and ‘hospital’ to capture further potential studies. The search was executed on 01/10/2024 using Scopus, Medline, CINAHL and PsycInfo databases. We did not search the grey literature.

The final search terms were “code of ethics” OR “code of conduct” AND doctor OR physician OR clinician OR “medical practitioner” OR nurs* OR “health profession*” OR healthcare OR “health care” OR “pharmac*” OR “dentist” OR “midwi*” OR dieti* OR “occupational therap*” OR “paramed*” OR “physiotherap*” OR “physical therap*” OR “radiograph*” OR “psycholog*” OR “prosthetist” OR “orthot*” OR “orthop*” OR “speech therap*” OR “art therap*” OR “chiropod*” OR “podiat*” OR “clinical scien*” OR “audiolog*” OR “hearing aid dispen” OR “operating department” OR “speech and language therap” OR “health worker” OR “allied health” OR “hospital”.

### 2.3 Screening and Eligibility Criteria

Papers were screened in a two‐step process. Search results were first exported to Rayyan (Ouzzani et al. 2016) where a title and abstract screen was carried out. At least two authors screened each paper; disagreements were resolved by a group discussion. A second full‐text screen was then carried out. Each paper was screened by the lead author and a second author. Disagreements were again resolved with a discussion. When we refer to codes of ethics below, we refer to any study that self‐identified as comparing codes of ethics, regardless of their form or function, whether the code in question was aspirational or had regulatory power or any other notable differences. No geographic or time/date restrictions were placed on the search or eligibility criteria.

Studies were included if:

- They made a comparison between a code of ethics and at least one other document. This document could have been a code or a related document, such as an oath or a code of conduct.
- They made a comparison where at least one group was health professionals.
- The full text was available and in English.

Studies were excluded if:

- They compared policy documents, oaths or similar documents and did not contain a code of ethics.
- They made comparisons where no codes related to healthcare professionals existed.
- They were theoretical papers or literature reviews or did not have data or substantive analysis.
- No full text was available, or the paper was in a language other than English.

### 2.4 Data Extraction

Data were extracted from included papers related to the studies' year of publication, the profession(s) in question and the countries in which the comparison was being made. A brief summary of each study's findings was also noted. We did not extract information from the codes of ethics themselves (those which were referred to in the studies); we only extracted information from the included studies themselves. Data was extracted by RE and checked by all authors. A summary of this is included in Table 1.

**TABLE 1:** Data extraction table.

Authors	Title	Year	Country	Discipline	Description
Bekemeier and Butterfield	Unreconciled inconsistencies: a critical review of the concept of social justice in 3 national nursing documents	2005	US	Nursing	This study compares social justice in three US nursing documents including the code of ethics
Berkman et al.	Gaps, conflicts and consensus in the ethics statements of professional associations, medical groups and health plans	2004	US	Medicine	This study compares codes of ethics with physician group practices and health plans. It includes data from 38 organisations
Booth	Hands up all those in favour of ethics	1996	Multiple	Psychology	This study compares the Canadian and Irish codes of ethics for Psychologists
Borysowski et al.	Ethics codes and use of new and innovative drugs	2019	Multiple	Medicine	This study examines international and national codes from the US, Canada, Australia, New Zealand, the UK, Ireland, France and Germany, comparing what is said on the use of new/innovative drugs
Borysowski et al.	Ethics codes and medical decision‐making	2021	Multiple	Medicine	This study examined international and national codes from the US, Canada, Australia, New Zealand, the UK, Ireland, Germany, France and Norway, comparing what is said about decision‐making
Byrd	A comparative analysis of moral principles and behavioural norms in eight ethical codes relevant to health sciences librarianship, Medical informatics and the health professions	2014	US	Multiple	This study examines eight interdisciplinary ethical codes, comparing how they specify moral norms (autonomy, beneficence, non‐maleficence and justice)
Conti	An analysis of the changes in communication techniques in the Italian codes of medical deontology	2017	Italy	Medicine	This study compares eight versions of the Italian codes of medical deontology, published from 1947 to 2014
Crisan and Iacob	Romanian code of pharmaceutical deontology: A new conception	2018	Multiple	Pharmacy	This study reviews numerous codes adopted by pharmaceutical bodies with the intent of developing a new code for Romania
Davey	Codes of ethics for laboratory medicine: Definition, structure and procedures—A narrative review based on existing national codes	2020	Multiple	Laboratory medicine	This study reviews several international laboratory medicine codes, seeking to provide guidance to national bodies in developing their own codes
Dobrowolska et al.	Moral obligations of nurses based on the ICN, UK, Irish and Polish codes of ethics for nurses	2007	Multiple	Nursing	This study reviews nursing codes from the UK, Ireland, Poland and the International Council of Nursing
Eriksson et al.	Do ethical guidelines give guidance? A critical examination of eight ethics regulations	2008	Multiple	Multiple	This study compares a eight international and interdisciplinary codes, related to the ethical guidance each provides
Essex et al.	Political action in nursing and medical codes of ethics	2024	Multiple	Multiple	This study compares 217 codes of medical and nursing codes to examine the content they include about political action
Gollust and Dwyer	Ethics of clinician communication in a changing communication landscape: Guidance from professional societies	2013	US	Medicine	This study compares codes of ethics and other documents from cancer‐related professional societies related to clinician communication
Haddara and Lingard	Exploring the premise of lost altruism: content analysis of two codes of ethics	2017	Multiple	Medicine	This study compares constructions of altruism is versions of Canada's medical code from 1868 to 2004
Hadjistavropoulos et al.	Ethical Orientation, Functional Linguistics and the Codes of Ethics of the Canadian Nurses Association and the Canadian Medical Association	2002	Canada	Multiple	This study compares the ethical orientation and linguistic differences between the Canadian nursing and medical codes
Holden	Exploring the evolution of a dental code of ethics: a critical discourse analysis	2020	Australia	Dental	This study examines two versions of the NSW branch of the Australian Dental Association (2012–2018)
Holden	What do dental codes of ethics and conduct suggest about attitudes to raising concerns and self‐regulation?	2018	Multiple	Dental	This study reviews dental codes across a number of countries, exploring their attitudes toward self‐regulation and content more generally
Leach et al.	Psychological ethics codes: A comparison of 24 countries	1997	Multiple	Psychology	This study examined 19 codes that represent 24 countries, exploring similarities and differences between countries codes
Leach et al.	Ethics Standards Impacting Test Development and Use: A Review of 31 Ethics Codes Impacting Practices in 35 Countries	2007	Multiple	Psychology	This study examined 31 codes that represented 35 countries, specifically exploring what they say about test development and use
Lewis et al.	Toward a national psychology ethics code: Systematic analysis of Australian professional and registration board standards	2014	Australia	Psychology	This study examined five state based and one national code of ethics for psychologists in Australia, comparing coverage of ethical standards
Malloy et al.	The codes of ethics of the Canadian Psychological Association and the Canadian Medical Association: Ethical orientation and functional grammar analysis	2002	Canada	Multiple	This study compares the Canadian Psychological and Medical codes
Milligan et al.	Achieving cultural safety for Australia's First Peoples: a review of the Australian Health Practitioner Regulation Agency‐registered health practitioners' Codes of Conduct and Codes of Ethics	2021	Australia	Multiple	This study examines cultural safety across the codes of the 16 regulated health professions in Australia
Parsonson	International Psychology Ethics Codes: Where is the “Culture” n Acculturation?	2018	Multiple	Psychology	This study examines the US psychology code against several international codes
Ricou et al.	Ethical principles in psychotherapy within a broad psychological and medical deontological framework: An international comparison	2023	Multiple	Psychotherapy	This study examines multiple codes related to psychotherapy, comparing their ethical content and principles
Rochon and Williams‐Jones	Are Military and Medical Ethics Necessarily Incompatible? A Canadian Case Study	2016	Canada	Medicine	This study examines military and medial ethics in Canada, comparing their content and identifying conflicts
Rothstein	A proposed revision of the ACOEM code of ethics	1997	US	Occupational and Environmental Medicine	This study examines a number of codes to inform the development of a new code for the American College of Occupational and Environmental Medicine
Rutkowska	Ethical dilemmas in online psychotherapy—A review of selected ethical codes and recommendations of psychotherapeutic and psychological associations	2022	Multiple	Psychology	This study examines 52 codes of ethics for psychologists in relation to working with patients remotely
Sawyer	Nursing code of ethics: An international comparison	1989	Multiple	Nursing	This study examines 19 nursing codes of ethics to compare their content
Saxen	Same Principles, Different Worlds: A Critical Discourse Analysis of Medical Ethics and Nursing Ethics in Finnish Professional Texts	2018	Finland	Multiple	This study examines the Finnish medical and nursing codes, to compare how each profession was culturally constructed in Finland
Sinclair	Developing and revising the Canadian Code of Ethics for Psychologists: key differences from the American Psychological Association code	2020	Multiple	Psychology	This study examines the US and Canadian psychology codes, discussing their key differences
Tisdale and Symenuk	Human rights and nursing codes of ethics in Canada 1953–2017	2020	Canada	Nursing	This study examines nursing codes of ethics and their evolution between 1953 and 2017
Valderama‐Wallace	Critical discourse analysis of social justice in nursing's foundational documents	2017	US	Nursing	This study examined US nursing documents, including the code of ethics, to explore how they conceptualise social justice

### 2.5 Data Synthesis

Given the nature of the papers included and the above research question, we utilised a narrative synthesis. This approach was well suited to our research question, offering us flexibility in how we analysed and arranged our results (Dixon‐Woods et al. 2005).

## 3 Results

### 3.1 Search Outcome

**FIGURE 1:**
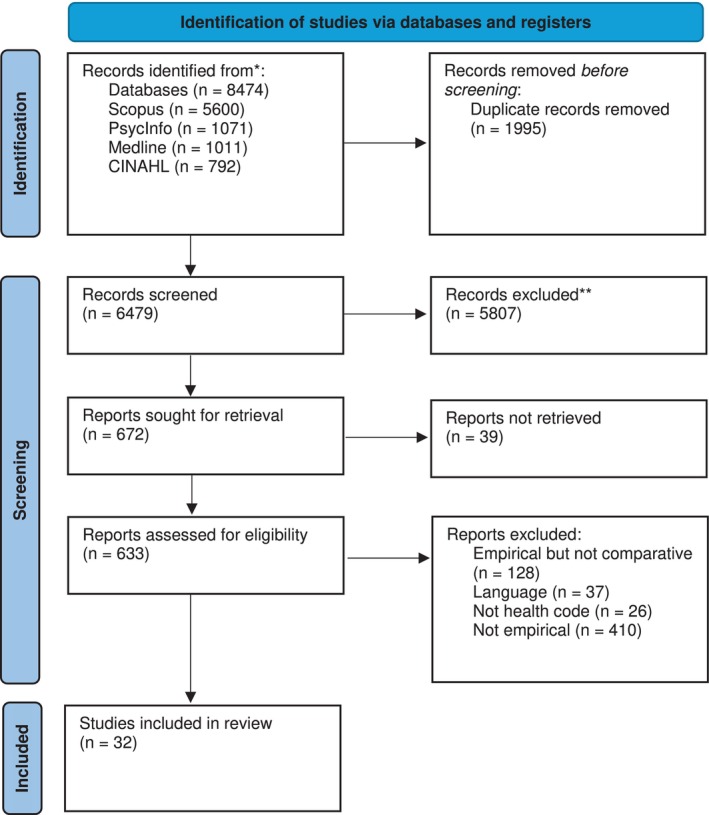
PRISMA 2020 flow diagram for new systematic reviews which included searches of databases and registers only.

The search yielded 8474 results. This was reduced to 6479 after duplicates were removed. A title and abstract screen was carried out on these papers, leaving 672 papers. A full‐text screen was then carried out, leaving 32 papers, which were included in this review. A summary of this process is outlined in a PRISMA flow diagram (Figure 1).

### 3.2 Descriptive Results of Included Studies

The studies included in this review represent a diversity of approaches, making comparisons within and between professions, as well as temporospatial comparisons; studies involved anywhere between two to 217 codes or documents. Amongst the studies, the majority made a comparison across multiple countries (*n* = 17), followed by studies in Canada (*n* = 4), USA (*n* = 7), Australia (*n* = 3), Finland (*n* = 1) and Italy (*n* = 1). Amongst the professions represented, most studies focused on medical codes (*n* = 7), and interdisciplinary comparisons (*n* = 7), followed by Psychology (*n* = 7), Nursing (*n* = 5), Dental (*n* = 2), Laboratory medicine (*n* = 1), Pharmacy (*n* = 1), Psychotherapy (*n* = 1), environmental and occupational medicine (*n* = 1). These results are summarised in Table 1.

The below discussion will be structured around these divisions, namely the profession(s) that the study in question compared. Within each group of studies, consideration will be given to the form (i.e., the structure of the code, its contents, principles or rules, for example) and function (what the code says it does (either explicitly or implicitly) of the codes being compared).

### 3.3 Medical Codes

Seven studies that compared medical codes of ethics. Two studies explored temporal changes. Haddara and Lingard (2017) for example explored how altruism was constructed in the Canadian and Australian medical code between 1868 and 2004. Within this timeframe, 19 codes were produced by the Canadian Medical Association, and 11 were produced by the Australian Medical Association. This study examined altruistic statements within these codes, identifying 17 statements in total. The authors concluded however, that this was shifting over time, with fewer altruistic statements in more recent versions of the code. Employing a similar temporal focus, examining Italian codes since the Second World War, Conti (2017) explored the ‘medical‐linguistic choices’ of the Italian medical code. In contrast to the paper above, this study found a number of new terms and concepts introduced in more recent codes, such as term ‘transplant’, ‘genetic’ and ‘sport’. Terms also varied over time, the terms ‘legal’ and ‘consent’ had increased in usage. Interestingly this study also tracks differences related to the terminology used to address patients over time. This study identified seven terms here: sick person, client, patient, person, citizen, subject and individual, with citizen, patient and individual the most frequently used words in more recent codes. While Haddara and Lingard (2017) suggest that ‘social and political’ factors influenced the inclusion of altruistic statements, both of these studies largely fail to engage with the reasons why certain terms have gained prominence or otherwise, so while they identify a shift over time, we can only speculate why this was the case here.

Like the study by Conti (2017) above, two further studies offered broad comparisons of codes, albeit with a cross‐sectional focus. Notably, each of these studies compared codes to other documents. Rochon and Williams‐Jones (2016) compared the Canadian Medical Association code of ethics to the Canadian Department of National Defence and Canadian Forces (DND and CF) Code of Values and Ethics. The authors argue the respective content of each of these codes related to values and principles are to a large extent, compatible. In saying this, however, there were some notable differences, t the most obvious being the focus of the codes, with differences present due to ‘choices regarding the core values and principles of the two professions: the CMA code is more focused on duties and responsibilities, while DND and CF code is focused on core values’. There were also notable differences when it came to autonomy, with those in the military having far less because of their position in a hierarchical organisation. This study raises questions about the adaptability of ethical codes across professions and contexts with fundamentally different power structures. While vastly different, Berkman et al. (2004) also compared codes to external documents; more specifically the US medical code was compared with ‘ethics policies at physician group practices and health plans, using the 1998–1999 policies of 38 organisations—18 medical associations (associations), nine physician group practices (groups), and 12 health plans (plans)’. This study focused on what the authors argued were three core ethical obligations—toward patients, toward resource allocation and toward society, raising more fundamental questions about what we should expect from codes and other related documents in regard to their scope and content. There were differences between the obligations specified in each document in relation to patients, with the authors finding that while professional associations documented obligations toward patients, such as beneficence and non‐maleficence, this was rarely if ever, done in health plans and practice policies.

Three further studies focused on the presence or absence of more specific content in medical codes. Gollust and Dwyer (2013), for example, explored 46 documents from 23 cancer‐related professional societies in the US, which included 12 codes of ethics. They explored the content in these documents related to communication. They found that while a number of these documents provided communication guidance, they generally focused on ethical issues related to patients and clinicians and said less about the ‘population or policy impact of communication’. Similarly, Borysowski et al. (2019) examined what international and national medical codes (from the USA, Canada, Australia, New Zealand, the UK, Ireland, France and Germany) said about new and/or innovative drugs with uncertain safety and efficacy. Out of the four international codes and guidelines analysed, only the Declaration of Helsinki addressed the question of the use of unproven drugs. Amongst national codes, only two (USA and New Zealand) explicitly allowed for the use of new or innovative drugs. In a more recent study, Borysowski et al. (2021) examined what codes said about medical decision‐making, notably whether the code could be considered to be promoting informed decision‐making, shared decision‐making or paternalism. This study focused on the World Medical Association code and several other national codes from the USA, Canada, Australia, New Zealand, the UK, Ireland, Germany, France and Norway. This study found that while the WMA code does not provide any specific model related to decision‐making, 10 of the 11 national codes contain guidance about informed decision‐making, while only four refer to shared decision‐making. Some codes contain articles, which are imprecise with regard to the question of medical decision‐making. While this study, like those above, draws attention to these differences, far less attention is given to placing them in social and historical contexts and exploring why these differences exist.

### 3.4 Interdisciplinary Studies

Four studies that made interdisciplinary comparisons explored differences between medical and nursing codes, while one compared medical and psychology codes. Hadjistavropoulos et al. (2002) sought to compare the codes of ethics of the Canadian Nurses Association (CNA) and Canadian Medical Association (CMA) in relation to their grammatical and linguistic structures. This study found that the CNA code contained proportionately more statements that provided a rationale for ethical behaviour and that statements in the CMA code tended to be more dogmatic. Both codes expressed a strong deontological tone; however, the CNA code promoted a more collaborative approach with patients, whereas the CMA code was more paternalistic. In another Canadian study, somewhat similar findings were reported when comparing the Canadian Psychological Association (CPA) and CMA's codes of ethics. Malloy et al. (2002) found that the CPA code had ‘greater educational value, is less authoritarian, provides a clear rationale for ethical behaviour, and is more empowering to the decision‐maker’ when compared to the CMA code. While each of these studies highlights important differences and while these are put down to ‘fundamental attitude differences between physicians and psychologists’ Malloy et al. (2002), it remains somewhat unclear as to whether these differences reflect substantive ethical commitments or merely differing rhetorical strategies to convey professional legitimacy. The above studies also appear to be based on assumptions about what a ‘good’ code should be, including the extent of guidance it should offer. Saxén (2018) also examined the differences between the nursing and medical codes in Finland. This study found what was described as a ‘cultural gap between the ethics discourses of medicine and nursing’, not dissimilar to the findings above. In the largest study included here (in terms of the number of codes included), Essex et al. (2024) examined 217 codes of medical and nursing ethics from around the world to examine the content they included about political action. This study drew temporospatial and professional comparisons across codes and concluded that while ‘there were noticeable differences between medical and nursing codes, overall, advocacy, activism and even politics were rarely discussed explicitly in most codes. When such action was spoken about, this was often vague and imprecise, with codes speaking of ‘political action’ and ‘advocacy’ in general terms’.

Two studies explored more specific issues across codes. Milligan et al. (2021) examined cultural safety related to Aboriginal and Torres Strait Islander peoples across the 16 health professions registered in Australia. This study found that only two adequately addressed cultural safety. The majority of codes conflated Aboriginal and Torres Strait Islander peoples with culturally and linguistically diverse communities. Of the 11 professions with a code of ethics, only Pharmacy and Psychology outlined specific ethical responsibilities to Aboriginal and Torres Strait Islander peoples. A further study here compared several codes external to healthcare, including those from a number of libraries compared to those from the American Medical Association, American Nurses Association and American Public Health Association. This study was motivated by the common issue shared across these professions related to health information management (Byrd 2014). Codes were compared against Beauchamp and Childress's ethical framework/principles (Beauchamp and Childress 2001), considering what codes said about autonomy, beneficence, non‐maleficence and justice. Unlike the articles above, this article spoke about the common ground found between these codes, with the authors pointing to the many opportunities for effective communication and collaboration in this area. While this study identifies a number of commonalities, it has a number of shortcomings in that it largely overlooks some of the more pressing concerns about principalism, including its application, not only in increasingly complex healthcare contexts but also how this might be applied across professions, as was done in this study.

A final study raised more fundamental issues for codes and the above critiques, questioning whether codes had any utility in relation to decision‐making at all. Eriksson et al. (2008) examined several guidelines, including the code of ethics from the ICN and the Swedish Medical Association. While these codes spoke in more general terms, the authors argue that they offer little in the way of specific guidance and that this points to a broader problem with codes more generally, namely that they do not guide action. This, of course, strikes at the heart of one of the more fundamental issues facing codes, notably the extent and specificity of the guidance they provide and whether they are, in fact, useful at all when it comes to clinical decision‐making.

### 3.5 Psychology Codes

Four studies provided broad comparisons of the content and structure of psychology codes. Lewis et al. (2014) carried out a review in the context of a structural shift in the registration of psychologists in Australia toward a national registration process and the adoption of a code of ethics. In this context, this paper sought to explore state and territory codes and the existing national code (already in place and authored by the Australian Psychological Society). This study concluded that the existing national code was more comprehensive and contained a greater number of ‘ethical standards’, a conclusion which, of course, does not necessarily mean the national code offered more ethically appropriate advice. In a larger study that used the American Psychological Society code as a benchmark, Leach and Harbin (1997) compared 19 codes from 24 countries to explore their general content. This study found that Canada's code was most similar to the US's and China's, which were the most dissimilar. Additionally, there were 10 ‘standards’ the authors identified as near universal, while there were eight that were unique to the US code. This study also spoke to how culture may impact codes and their content. Similar conclusions were drawn by Parsonson and Alquicira (2019) who also compared the US code to multiple international codes, concluding that codes should be viewed within the cultural context. While the above study found that the US and Canadian codes were most similar in comparison to other international codes, a study from Sinclair (2020), who only examined the US and Canadian codes, stands in comparison. This study found several key distinctions between the codes and argued that, amongst other differences, the Canadian code had a greater focus on ‘(a) the concept of a contract with society; (b) aspiration versus enforcement; (c) ethical reasoning and decision‐making; and (d) organizing standards around ethical principles’. One further study also compared the Canadian code for psychologists to that of Ireland; instead, however, this study examined the structure of the codes as they related to decision‐making (Booth 1996). The authors argue that the Canadian code ‘offers a clear and practical structure, even including guidelines for those situations when ethical principles are in conflict’ in comparison to the Irish code. The above conclusions that culture influences the similarity of codes is perhaps unsurprising; a key issue in the above studies however also appears to be that whether there were differences or similarities found between codes was dependent on the level of analysis and abstraction undertaken, with more focused studies like Sinclair's (2020) likely to identify greater differences where other studies did not.

Two further studies examined more specific content and its presence or absence in codes. Leach and Harbin (1997) explored what 31 codes representing 35 countries said about tests and testing instruments, again using the American Psychological Societies code as a ‘benchmark’. This study found that about one‐third of the codes included here did not address test use. Amongst the codes that did, statements about the explanation of results, correct use of the test, and restrictions on tests for unqualified people were found more frequently, while statements about test construction and the use of obsolete tests were found less frequently. One further study that explored codes for more specific content was prompted by the COVID‐19 pandemic and the sudden shift to online working. Rutkowska (2022) examined 52 codes of ethics to explore what was contained in relation to online working with patients. Again, results were mixed; while some codes had guidance for online working, many did not even after 2 years into the pandemic.

### 3.6 Nursing Codes

Three studies related to nursing codes made comparisons as they related to specific content: social justice and human rights. Tisdale and Symenuk (2020) explored how human rights were conceptualised in Canadian codes from 1953 to 2017. This study found there had been very little change to how human rights have been represented in codes. Two further studies examined social justice in the ANA code. Bekemeier and Butterfield (2005) explored a number of nursing documents, including the code of ethics, arguing that across these documents, social justice was conceptualised in ‘an inconsistent, ambiguous, and superficial’ way. Similar findings were presented by Valderama‐Wallace (2017) who also examined US nursing documents and found inconsistencies in how social justice was conceptualised.

Two further articles had a more general focus. Dobrowolska et al. (2007) compared the ICN code and codes from the UK, Ireland and Poland. The findings of this study suggest that in terms of content, ‘many of the nurses' moral duties mentioned in the four codes are the same’. There was a notable difference, however, related to the specificity of these codes. Sawyer (1989) analysed codes of 19 national and two international nursing associations to examine their content. This study found that across all codes, most failed to deal with particularly contentious issues, with some failing to engage with ethical issues at all, instead focusing on the commercial aspects of practice. While these findings perhaps also reflect the tensions in nursing being both a caring profession and a workforce often embedded in market‐driven healthcare systems, they are reflected in a far more recent study already noted above, that found that not only nursing but medical codes often failed to provide guidance about advocacy and other forms of political action (Essex et al. 2024).

### 3.7 Other Disciplines

Amongst the other codes included in this review, two related to Dentistry and the remaining four to Laboratory Medicine, Pharmacy, Psychotherapy and Occupational and Environmental Medicine, respectively.

Holden (2018) examined 17 dental codes of ethics, representing 12 countries, Europe and the World Dental Federation. This study sought to explore self‐regulation in these codes. The results of a thematic analysis revealed four themes: ‘(1) explicit expression of the need to report; (2) warning against frivolous reporting; (3) acceptance of reporting being difficult and; (4) threshold requiring a professional to report’. The findings of this study show that while these codes contained content related to self‐regulation, this was often ‘accompanied by an anxiety surrounding unsubstantiated or malicious reporting’. In a further study, Holden (2020) also examined Australian dental codes, specifically the code of ethics produced by the New South Wales Branch of the Australian Dental Council. This study sought to examine ‘contradictions between the discourses within the codes and how these relate to broader social realities that surround the dental profession’ in the 2012 and 2018 codes. Not dissimilar to the above findings, this study found that these codes failed to ‘consider the public as a key stakeholder in the creation and curation’ of the code, suggesting that ‘both codes amount to declarations of professional privilege and dominance’. Holden concludes that this showed ‘that the current code of ethics is still reluctant to recognise and engage with the public as an equal stakeholder in the planning and provision of oral health care and the development of the profession's values and cultural trajectory’.

One study examined laboratory medicine. In this study, Davey (2020) sought to explore three international codes (US, Poland and Australasia) to provide ‘guidance at the level of definition, structure and procedures to assist national societies, and their clinical chemistry and laboratory medicine professionals in the task of crafting their own Ethics Code’. The results of this study are largely uncritical and speak to the ways in which codes vary while expressing similar principles, such as a duty to the patient, colleagues and society more generally. Similarly, Crişan and Iacob (2018) sought to examine international pharmacy codes to inform a revision to the Romanian Pharmaceutical code. Like the above study, this study is largely uncritical, identifying common and shared principles amongst codes to inform the new Romanian code. One final study that sought to compare other codes as they related to the development of a new code came from Rothstein (1997), who compared codes of the American College of Occupational and Environmental Medicine. This study examined the appropriateness of international and other codes and their adaptation to the US context. While critical of these codes, this study offers few insights when it comes to the comparison of the codes themselves.

Perhaps most closely related to the above studies that examined psychology codes, one study examined codes for psychotherapists. In this study, Ricou et al. (2023) explored 61 codes related to psychotherapists. The most common principles shared amongst these codes were confidentiality, competence and integrity. The authors note that the codes contain little about broader social responsibilities or obligations.

## 4 Discussion

The above review sought to map and synthesise the literature on comparative studies in relation to codes of ethics and, in particular, their form (i.e., the structure of the code, its contents, principles or rules for example) and function (what the code says it does, either explicitly or implicitly) and points of con/divergence. Notably, differences were seen across professions, countries and time, suggesting that culture, history, politics and perhaps even geography influence the content of codes. Some patterns are worth noting, which may be particularly fruitful areas for future research. A number of studies noted differences between medical and nursing codes, speaking to a ‘cultural divide’ between the professions when it came to ethics. This is not unique to healthcare. If we look further afield to public service codes of ethics, we glimpse similar issues. In a content analysis of seven codes of ethics from professions commonly found in public service, Odeh (2024) found a type of cultural divide in what she refers to as ‘low‐road versus high‐road ethics’ (p. 153). Low‐road ethics involves behaving in a way that does not break the law and focuses on compliance and avoidance misconduct. High‐road ethics, in contrast, is aspirational and promulgates concepts of fairness and justice. In regards to nursing and medical ethics, there has been a longstanding discussion about the nature and extent of the differences between nursing and other forms (i.e., medical and bio) of ethics (Pilkington and Giuliante 2023). Codes of ethics have the potential to offer a number of insights here and to inform these debates.

Studies also gave insight into how codes changed over time. Some terms, such as human rights, at least in the Canadian nursing code, remained constant over time. Other terminology (like that related to altruism in Canadian and Australian medical codes) has become less used, with others becoming increasingly used (like legal terms in the Italian medical code). While the fact that codes change is well established and seems somewhat unsurprising, what is often missing from these accounts are the factors that led to these changes: social, political, professional or otherwise. Future studies should place greater emphasis on attempting to map these factors. That is, why codes differed across time, place and profession and what contributed to these similarities or differences. Related to these shifts over time, the above results also point to critical social and political events as shaping the content of codes. At least one study examined the content of codes and their advice around online working in light of the COVID‐19 pandemic. This raises questions over the extent to which codes should adapt, particularly to such events. As noted in the introduction, revising codes in light of social and political upheaval has not always been for the better.

Studies also point to more fundamental issues as they relate to the meaning of concepts and guidance. At least two studies suggested that social justice was defined inconsistently across US nursing documents, for example. While there is clearly value in having clarity and well‐defined concepts, we feel that inconsistencies should not always be judged as a problem, nor should concepts be judged against pre‐specified criteria. Understandings of rights and duties in relation to health and healthcare will inevitably vary throughout the world. Some codes may use similar terminology but may mean something slightly different. There seems to be potential here to explore the more implicit meanings attached to certain concepts used throughout different codes.

Even where there is agreement on the concepts included in codes, several further challenges arise, namely in how these concepts are presented, along with the breadth and specificity of the code itself. Throughout a number of studies included above, there appeared to be an assumption (that was sometimes made explicit) that codes, when presented a certain way, whether this be with greater specificity or detail, were better. In their study on researchers' perceptions of ethical codes of conduct, Giorgini et al. (2015) found that when codes were clear, consistent and laid out definitive courses of action, they were used. However, in the absence of clarity, people defer to professional norms or rely on their existing morals. The obvious problem here is that professionally accepted norms or personal beliefs may be incongruent with the best course of ethical action. While this may lead us to conclude that more comprehensive codes are better or necessary to guide ethical behaviour, this is not the case. Dawson (1994) for example, argues that codes are limited in at least two ways: they cannot account for future conflicts, nor can they fully resolve situations where principles conflict. We could, of course, continually revise codes to address each new issue, but greater detail and complexity also come with greater potential for conflicting principles, and in the end, we are not left with anything that resembles the code at all. An alternative to this is what Dawson (1994) calls a ‘cognitivist’ account of ethics, notably that instead of relying on rules, we empower people to respond in an ethically flexible manner to unforeseen and conflicting circumstances.

While this study appears to have only complicated and problematised the use of codes (and while this is true to some extent), we still think there are several useful and more practical takeaways from the above analysis. First, care is needed in ‘benchmarking’ or in assuming that one code is superior to another, put another way, our expectations of codes will inevitably inform what it is we think they should ‘do’ and for this, there appears to be one right answer. Second, and related to the above point, variation and difference are not necessarily bad things; they are things to be mindful of and may require careful negotiation. Third, we feel that comparative studies will become increasingly important as efforts are made to decolonise health and healthcare. Codes, even with their shortcomings, remain important documents if for no other reason other than setting aspirational goals for the professions. Their structure and content should be critically examined, particularly where international codes are being adopted. Finally, from a teaching perspective, comparative analysis of codes could offer valuable insight into how ethics differ across time, place and profession, offering student's insight into how ethics differ and providing them with greater ethical flexibility to navigate and negotiate ethical challenges.

More recent debates were had about whether codes should be changed in light of the COVID‐19 pandemic (Komparic et al. 2023). Only time will tell how the pandemic shapes codes into the future, if at all. Taking a longer timeline than what was provided in the above studies (which stretch back about three decades), we see more dramatic shifts in codes and ideas about ethics more generally. Baker (2012), for example, speaking about oaths and ethics more generally, notes, ‘fidelity to the sick person was not an enduring feature of medical ethics from ancient times to the present day … historically, medical oaths sworn by practitioners, even in the Anglo‐American cultural sphere, placed loyalty to crown/state, or to the church, above fidelity to the sick person’. We can only speculate as to what future changes codes will undergo; however, with healthcare becoming more fused to artificial intelligence, we foreshadow its corresponding dominance in the codes of the future. |In this regard, perhaps healthcare codes of the future won't just apply to humans. Ma et al. (2024) describes aspirational ethics for human‐bot psychoanalysis—designed to guide large language models on how to act in the event of a crisis, amongst other things—illustrates this point.

There are a number of limitations to the above review worth mentioning. First, because of the nature of this review, we have not included theoretical papers here. There is a rich theoretical literature that offers a number of insights as they relate to codes; future work should attempt to integrate these perspectives. Secondly, and related to this point, this paper includes little about the nature of codes, their development and history. A differently scoped review would address this; there appears to be substantive value in looking to history to better understand codes today. Finally, this review did not review the codes of ethics themselves. It would be a huge undertaking to examine potentially hundreds of codes, their content and function. Future research should attempt to synthesise codes themselves to better understand their functions, similarities and differences.

This review sheds light on the vastly different nature of codes and how these differences manifest across time and place. While there are a number of similarities in codes, there are multiple places of divergence, with many only sharing a similar title and a little more. There is much to be gained from further comparative studies on codes, as such work could help us better understand the potentials and pitfalls of these documents and their evolution, from past codes into the future.

## Author Contributions

R.E. conceived this work and led screening, extraction, analysis and write‐up. All other authors assisted with screening, extraction and in drafting this paper.

## Ethics Statement

The authors have nothing to report.

## Consent

The authors have nothing to report.

## Conflicts of Interest

The authors declare no conflicts of interest.

## Supporting information


Appendix S1.

## Data Availability

Data for this paper is publicly available.

## Endnotes

^1^
For example, the ethical assumptions embedded within codes.
